# Neural Network-Based Hammerstein Model Identification of a Lab-Scale Batch Reactor

**DOI:** 10.1021/acsomega.3c05406

**Published:** 2023-12-21

**Authors:** Murugan Balakrishnan, Vinodha Rajendran, Shettigar J. Prajwal, Thirunavukkarasu Indiran

**Affiliations:** † Department of Electronics and Instrumentation Engineering, Annamalai University, Annamalainagar 608 002, Tamil Nadu, India; ‡ Department of Electronics and Instrumentation Engineering, Annamalai University, Annamalainagar 608 002, Tamil Nadu, India; § Department of Mechatronics, Manipal Academy of Higher Education, Manipal 576 104, Karnataka, India; ∥ Department of Instrumentation and Control Engineering, Manipal Institute of Technology, Manipal Academy of Higher Education, Manipal 576 104, Karnataka, India

## Abstract

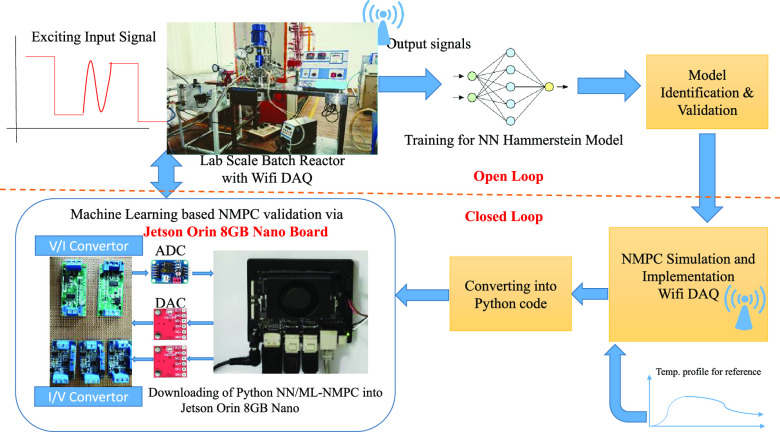

This paper focuses on two types of neural network-based Hammerstein model identification methods for the acrylamide polymerization reaction of a batch reactor process. The first neural-based identification type formulates the weights of the multilayer network directly as parameters of the nonlinear static and linear dynamic blocks of the Hammerstein model and trains the weights using a gradient-based backpropagation algorithm. In the second identification type, the nonlinear static block of the Hammerstein model is framed as a single hidden-layer feedforward network and both nonlinear and linear block parameters are trained using an extreme learning machine, where the training procedure is exempted from gradient calculation. The primary focus of the paper is neural-based model identification of a complex nonlinear system, which facilitates ease of linear/nonlinear controller design with good learning speed and less computations. A future work toward the machine learning-based nonlinear model predictive controller implementation using the Jetson Orin Nano board is also described.

## 1 Introduction

Due to exothermic reactions, reaction constants with exponential terms, and the sort of polymerization reaction that takes place in the reactor, batch reactors are essentially considered as a highly nonlinear process.^1,2^ In a batch process, the feed is only charged once and held at the same quantity until the batch process is finished. There is neither a continuous feed inflow nor a continuous feed outflow. Only after the batch process terminal time is reached, the product is removed for analysis. There are a few minor external disturbances that can affect how a batch reactor operates. These include environmental changes, variations in the temperature of coolant flow rate over time, and jacket and reactor temperature variations (*T*_j_ and *T*_r_). By examining the batch reactor’s and continuous stirred tank reactor nonlinear differential equations^1−3^ as addressed by Shettigar et al.^1,2^ and Peng et al.,^6^ which are provided under system dynamics, the aforementioned can be realized. The batch reactor issue can be split into two categories: (i) model identification and (ii) closed-loop control operation of a batch reactor with the formation of an ideal temperature trajectory.

This work addresses the first issue of a batch reactor. Nonlinear model identification is a challenging task for researchers. Compared to the black-box model of a nonlinear system, Wiener and Hammerstein models, which are defined as combination of static nonlinear block and linear dynamic block, find a physical relation with the original nonlinear system. Neural network models are capable of approximating highly nonlinear system dynamics but have no physical relation with actual nonlinear systems, if done by black-box modeling. However, neural-based Wiener^6^ or Hammerstein^5^ models have clear model structure and model parameters, hence well related with actual nonlinear systems. As a result, the first issue of batch reactor model identification is approached by two different neural-based Hammerstein structures in the proposed work.

The nonlinear structure identification in the Hammerstein model is approached by either parametric or nonparametric identification procedures. In parametric identification, the nonlinear function is formulated as a finite order polynomial and unknown coefficients to be extracted.^7^ In nonparametric identification, the nonlinear static function is found to be continuous function series like orthogonal function,^8^ polynomial function,^9^ block pulse function,^10^ etc. Neural and fuzzy systems are used in the identification of nonlinear static blocks as they have the universal approximation ability of nonlinear functions. However, here the steady state and transient data of the plant is essential to identify static nonlinear block from linear dynamic block. In addition, MLP, RBFNN and B-spline Neural Network, zero TS fuzzy model, etc. are used to identify a nonlinear block of the Hammerstein model.

In the first model identification approach, both the nonlinear static block and linear dynamic block of the Hammerstein model^11^ are entirely formulated as a multilayer neural network. The weights of the thus formulated network are equivalent to parameters of the Hammerstein model, and a gradient-based weight updating procedure is followed to reduce the error between predicted output and actual output.

Even though the above-mentioned neural-based identification procedure of the Hammerstein model provides satisfactory results, a certain amount of complexity is involved like gradient calculation in the multilayer network.

In the second type of identification, exploiting the universal approximation ability of neural networks and avoiding the complexity of finding gradients, an effective training algorithm for a single hidden-layer feedforward network (SLFN) was proposed by Huang et al.^12^ called an extreme learning machine (ELM). The algorithm was found to be simple and widely used in regression and classification problems. The ELM algorithm defines the identification of weights that are connecting the hidden layer to the output layer using least-squares estimation by randomly fixing weights and biases of the input layer to the hidden layer. Despite the simplicity in the learning algorithm, ELM has faster learning capability and good approximation of nonlinearity. Further, as backpropagation of weights is not involved in ELM, there is no need for choosing training parameters like learning rate, number of epochs, or stopping criteria.

The proposed work has the credit of neural-based model identification of a complex batch reactor process with clear partition of linear and nonlinear structure, which in turn helps in suitable linear or nonlinear controller design.

Some of the following recent literature suggests hybrid modeling, which is a combination of data-driven and first-principle modeling. Ammar et al.^13^ carried out the recurrent neural network-based modeling for the experimental reactor to predict the concentrations of four variables in the esterification reaction. The authors generated the database with the experimentation and then carried out the modeling for different initial conditions and ranges; validation of the identified model is also carried out by comparing the actual and predicted concentrations.

Jery et al.^14^ in their machine learning (ML) model of the batch reactor used a multilayer perceptron artificial neural network using linear, sigmoidal, ReLU activation functions for the prediction. The authors have normalized the data before processing for the modeling to improve the accuracy of the model. By iterating the hidden layers, activation functions and epoch errors are minimized.

Zhang et al.^15^ used the Koopman modeling for designing the predictive controller for a nonlinear reactor application. The authors have proposed the reduced order Koopman model predictive controller in comparison with the full order Koopman model predictive controller and proved that the computation time is less in the reduced order.

Tian et al.^21^ developed the Koopman model for the wastewater treatment plant for the accurate prediction of the anaerobic-anoxic-oxic data-driven model to overcome the limitations of the general data-driven model, which are not more accurate in case of dynamic variations in the external environmental changes and stochastic perturbations.

Gupta et al.^22^ presented the iterative learning control (ILC)—explicit model predictive control two-tier framework design for the batch reactor transesterification process for biofuel production in simulation. Authors have considered the multiple uncertainties in activation energy and input concentration of triglyceride. The forgetting factor is used in the ILC for the control moves to deal with the disturbance factor.

Norouzi et al.^23^ gave the overview on the timeline development of model predictive control. ML improves the optimization and computation time reduction in the MPC. Based on the specific application, the integration of ML with MPC needs to be integrated. Neural network-based MPC using embedded concept results with computation time effectively was highlighted.

## 2 Hammerstein Model

Generally, Hammerstein model is called the block-oriented model,^16^ since it has a static element for a nonlinearity and describes the dynamics with a linear model. In other words, the Hammerstein model is a linear filter acting on a nonlinearly transformed input. It is also equivalent to several output estimation models. When compared to the Weiner Model, the Hammerstein model is robust with respect to the input signal switching time. The Hammerstein model responds less to the perturbations on the steady-state output, compared to the Weiner model for the same input signal. Handling of the Hammerstein model is more flexible than the Weiner model since the quantitative behavior of the Hammerstein will not vary much for the varying magnitude signals.

**Figure 1:**
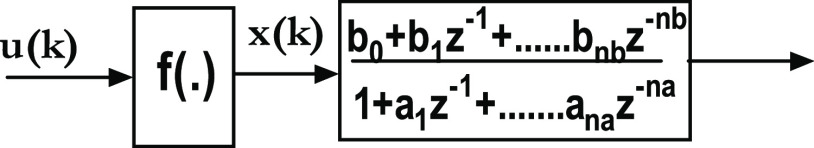
General Hammerstein model structure.

Hammerstein model is a mixture of static nonlinear function *f*(.) and a linear dynamic subsystem *G*(*z*^–1^) as shown in Figure 1

The mathematical representation of SISO Hammerstein model is represented as
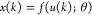
1
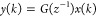
2

where *u*(*k*), *x*(*k*), and *y*(*k*) represent input signal, intermediate signal, and output signal, respectively. The linear dynamic subsystem is further expanded as
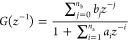
3

where *n*_a_ and *n*_b_ represent the order of denominator and numerator polynomial of linear subsystem with *n*_b_ ≤ *n*_a_.

Hence, the discrete form of SISO Hammerstein model is stated as
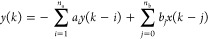
4

### 2.1 Multilayer Neural-Based Hammerstein Model

**Figure 2:**
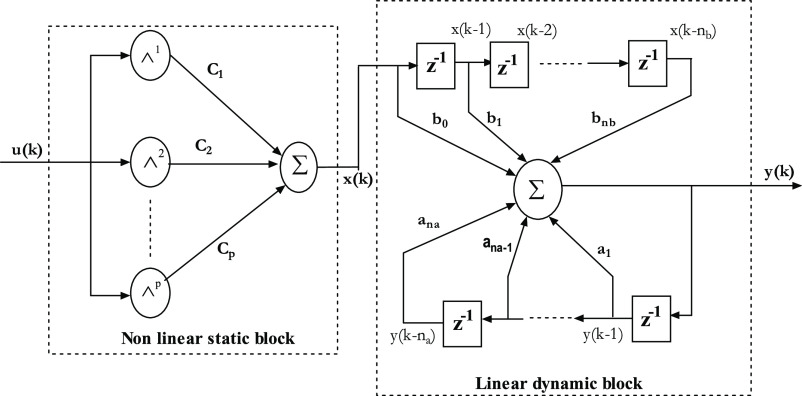
Multilayer neural-based Hammerstein model.

The multilayer neural network formulated as a nonlinear static block successively followed by a linear dynamic block of the Hammerstein structure is shown in Figure 2. The input, output, and unmeasured intermediate variables are defined as *u*(*k*), *y*(*k*), and *x*(*k*), respectively. The intermediate variable is defined as a weighted polynomial structure of input *u*(*k*) and the linear block is the ratio of the weighted delayed intermediate variable *x*(*k*) to the weighted delayed output variable *y*(*k*):
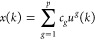
5
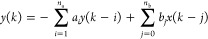
6

where *c*_*g*_ for *g* = 1, 2...*p*, *a_i_* for *i* = 1, 2,...*n*_a_, and *b_j_* for *j* = 0, 1, 2,...*n*_b_ are the weights of the network or parameters of the nonlinear and linear block of the Hammerstein system. Hence, the parameters of the Hammerstein model are the weights of the multilayer neural network, obtained by the backpropagation learning algorithm.

### 2.2 Learning Algorithm of the Multilayer Neural-Based Hammerstein Model

The identification of multilayer neural-based Hammerstein model parameters^11^ is achieved by minimization of squared error function *e*(*k*) given in eq 7:
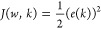
7

where *e*(*k*) = *y*(*k*) – *ŷ*(*k*); *y*(*k*) is the actual output and *ŷ*(*k*) is the neural predicted output. Here, *w* represents the weights of the multilayer network or parameters of the Hammerstein model:
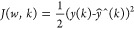
8
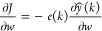
9

The weight update rule is given by eq 10
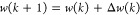
10
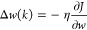
11

Substituting eqs 9 and 11 in eq 10, the update rule becomes
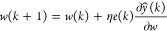
12

where 'η' is the learning rate, which will control the stability of the network and speed of convergence, and selection of 'η' is given by Peng et al.^6^

The update of parameters *a_i_*, *b_j_*, and *c_g_* as per eq 12 is given from eqs 13–21:
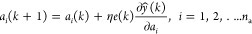
13

where
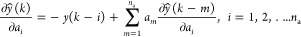
14
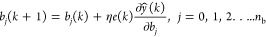
15

where
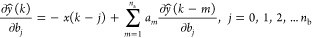
16
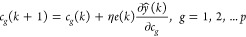
17

where
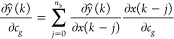
18

where
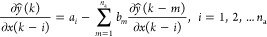
19

and
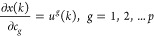
20

Substituting eqs 19 and 20 in eq 18:
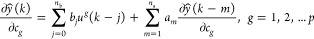
21

### 2.3 Extreme Learning Machine

**Figure 3:**
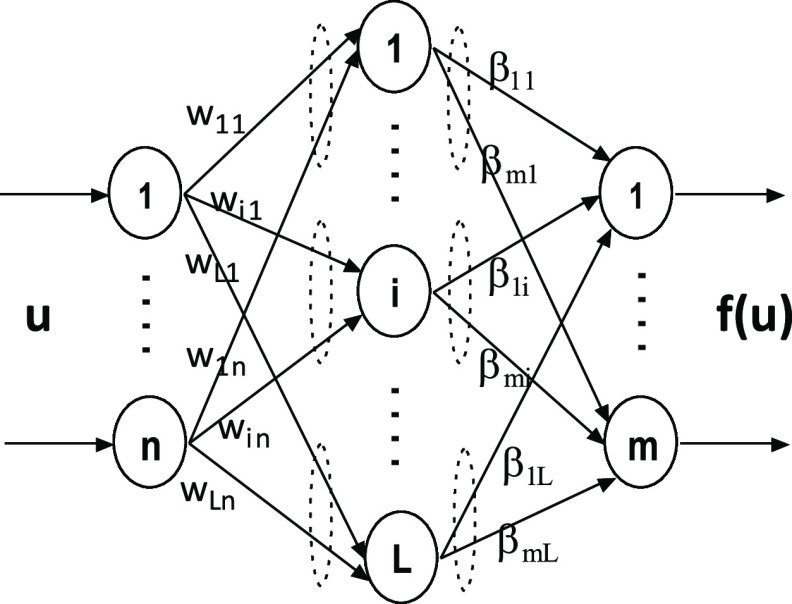
Single hidden-layer feedforward network (SLFN).

SLFNs form the basis for an ELM. It is a popularly used neural network approach to approximate a complex nonlinear function. The configuration of SLFN shown in Figure 3 has '*L*' hidden neurons as a single layer mapping of '*n*' input neurons to '*m*' output neurons. The input–output data set of length '*N*' is represented as *D* = {(*u*_*k*_, *y*_*k*_)|*u*_*k*_ ∈ *R*^*n*^, *y*_*k*_ ∈ *R*^*m*^, *k* = 1,2···*N*}. The output mapping of SLFN is represented mathematically as
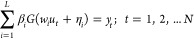
22

where *w*_*i*_ = [*w*_*i*1_, *w*_*i*2_,···*w*_*in*_] is the input weight vector passing '*n*' inputs to *i*th hidden neuron and β_*i*_ = [β_*i*1_, β_*i*2_,···β_*im*_] is the output weight vector passing *i*th hidden neuron information to '*m*' output neurons. The activation function of hidden neurons is denoted by '*G*' acting on the inner product of input weight vector *w_i_* and input *u_k_* with bias η_*i*_ ∈ *R* added to it.

The activation function is selected as the sigmoid function as given in eq 23:
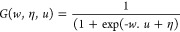
23

The defined SLFN approximates any nonlinear continuous function with zero error between predicted output '*O*' and actual target '*y*' as given in eq 24:
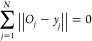
24

Then, there exist *w_i_*, β_*i*_, and η_*i*_, such that
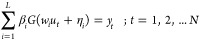


The compact form of '*N*' equations can be given as


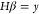
25

where *H*, β, and *y* are represented in matrix form as
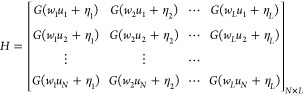
26


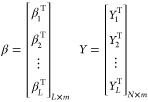
27

ELM algorithm^16^ states that appropriate activation function with randomly chosen input weight vector *w_i_* and bias vector η_*i*_ for training data *D* can give output weight vector β_*i*_, which is the least-squares estimate given in eq 28:
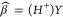
28

where *H*^+^ is the pseudoinverse as stated in the literature.^12^

### 2.4 ELM-Based Hammerstein Model

**Figure 4:**
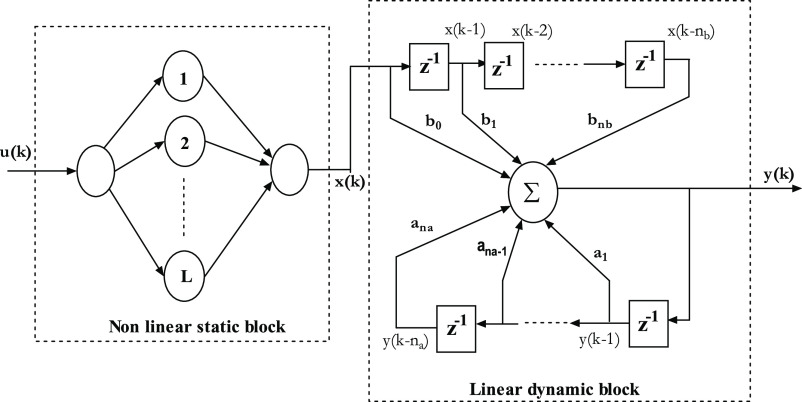
ELM-based Hammerstein model structure.

The ELM-based Hammerstein model shown in Figure 4 shows that the intermediate signal x(k) is viewed as output of SLFN trained via ELM.^17^ Hence, *x*(*k*) is given as
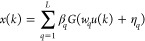
29

The output *y*(*k*) of the Hammerstein model is obtained by substituting eq 29 in eq 4:
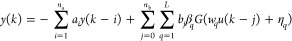
30

Formulating eq 30 as a linear regression problem with training data of '*N*' samples, the output *y*(*k*) is given as
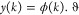
31

where *y*(*k*), ϕ(*k*), and parameter vector ϑ are given as
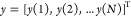
32
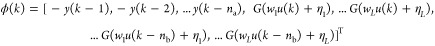
33
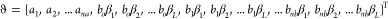
34

The parameter vector ϑ is identified as a solution of least-squares estimate of eq 31 by multiplying pseudoinverse of ϕ(*k*) and *y*(*k*):
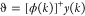
35

## 3 Batch Reactor

**Figure 5:**
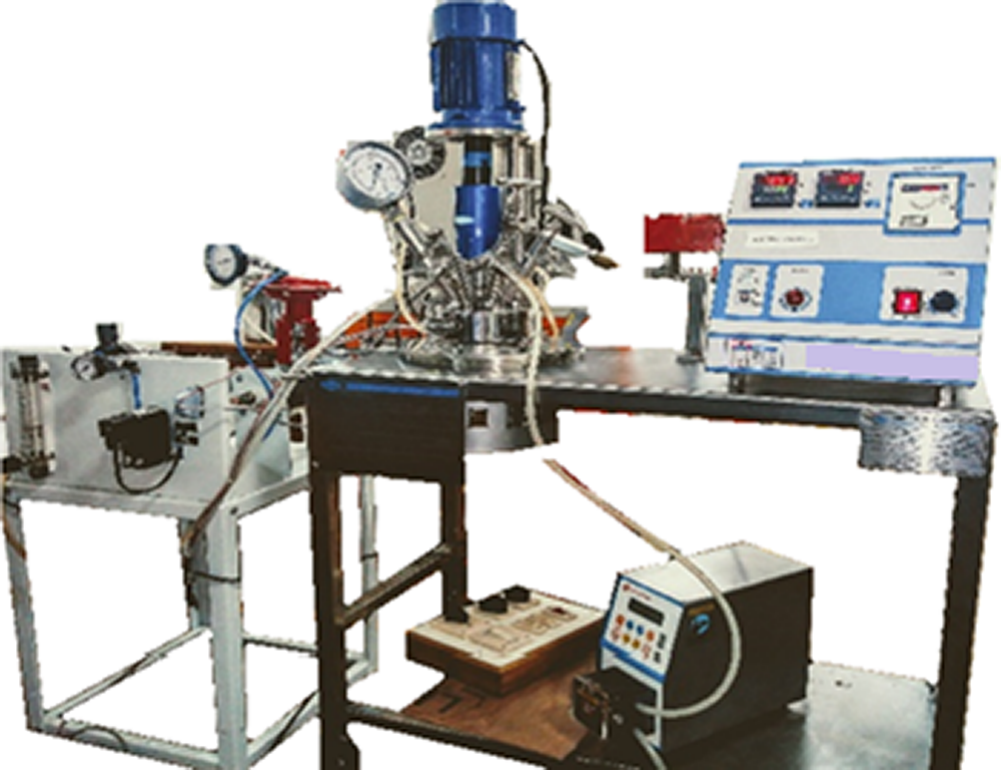
Lab-scale batch reactor setup in Research Lab-12 ICE Dept., MIT, Manipal.^1,2^

**Figure 6:**
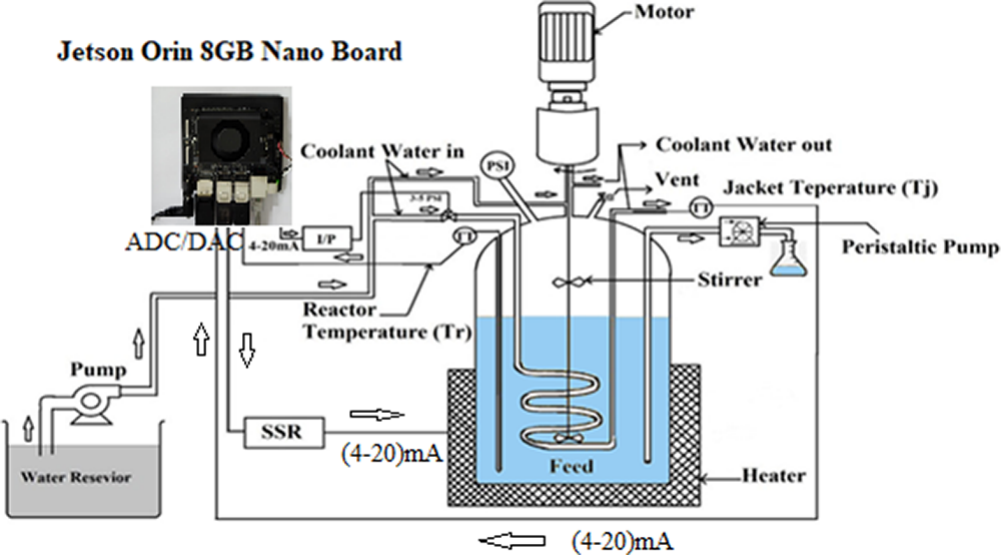
Schematic diagram of batch reactor setup with a standalone controller using Jetson Nano for NN/ML-NMPC validation available in MIT, Manipal.

The chosen batch reactor is exothermic and the reaction equation of the chemical feed and output is highly nonlinear.^2^ Additionally, it is a nonsteady state process, and its nonlinear dynamics are represented in the first-principle model as presented by Shettigar et al.,^1,2^ Peng et al.,^6^ and Yadav et al.^4^ The experimental batch reactor setup is illustrated in Figure 5 and its schematic model is given in Figure 6. The internal heating and cooling system of the custom batch reactor for the polymerization reaction help to maintain the desired reactor temperature. There is no physical contact between the heater and the reactor vessel when a ceramic band heater is employed to radiate heat into the vessel. This precaution has been taken to prevent the heater filament from corroding as a result of chemical interactions. Water is used as a cooling medium in this experimental effort. Heater current is maintained constant, and coolant water serves as a manipulated variable. A pneumatic control valve was used to set up a coolant flow station. Maximum coolant flow rate is limited to 0.75 LPM. Three RTDs are utilized in the experimental setup to detect the temperatures of the reactor (*T*_r_), jacket (*T*_j_), and coolant inlet water (*T*_c_). In a batch reactor, the chemical feed for the polymerization reaction is prepared and charged one at a time, and an initiator must be added right before the experiment is set to begin. The objective of the work is that real-time input–output data are collected for the chosen batch reactor process, and using a neural network as a base, two Hammerstein models are identified to fit the data.

## 4 Results and Discussion

This section presents the modeling expressions involved for the Hammerstein and ELM, along with its performance evaluation table. Model fit and its regression plots are also briefed.

### 4.1 Model Identification of Batch Reactor

Practical chemical systems are highly complex, and their nonlinear model identification is tough for any optimization methods. In the proposed work, real nonlinear dynamic of a batch reactor process is identified by two types of neural-based Hammerstein models. In both types, the underlying parameters and procedures are found to be the same. The linear block structure is limited using the Lipschitz quotient. 1000 data pairs {*u_i_*, *y_i_*}, respectively, the input feed rate and reactor temperature are used for identification of both the models.

### 4.2 Structure Determination for Linear Dynamic Block

The success of nonlinear system identification by Hammerstein modeling not only depends on suitable approximation of nonlinear static block but also needs proper structure selection of linear dynamic block or proper integer values of *n*_a_ and *n*_b_. Although literature^18^ guides many structure selection criteria, the present work follows Lipschitz quotient criteria^19^ proposed by He and Asada for proper order selection of linear dynamic block. Table 1 states Lipschitz quotient values for various inputs applied to the multilayer neural-based Hammerstein and ELM-based Hammerstein model.

**Table 1:** Lipschitz Quotient Values

Lipschitz quotient representation	inputs applied to the model	Lipschitz quotient '*q*' value (multilayer Hammerstein model)	Lipschitz quotient '*q*' value (ELM-based Hammerstein model)
*q*(0,0)	*u*(*k*)	infinity	infinity
*q*(1,0)	*y*(*k* – 1), *u*(*k*)	32.5436	0.9776
*q*(1,1)	*y*(*k –* 1), *u*(*k*), *u*(*k –* 1)	17.2371	0.9767
*q*(2,0)	*y*(*k –* 1), *y*(*k –* 2), *u*(*k*)	6.2332	0.7203
*q*(2,1)	*y*(*k –* 1), *y*(*k –* 2), *u*(*k*), *u*(*k –* 1)	5.4887	0.7118
*q*(2,2)	*y*(*k –* 1), *y*(*k –* 2), *u*(*k*), *u*(*k –* 1), *u*(*k –* 2)	5.4235	0.7028
*q*(3,0)	*y*(*k –* 1), *y*(*k –* 2), *y*(*k –* 2), *u*(*k*)	5.4233	0.7022

As per the optimal order selection criteria,^18^*y*(*k* – 1), *y*(*k* – 2), *u*(*k*), *u*(*k* – 1) are found to be the inputs for estimating *y*(*k*) in both the neural-based Hammerstein model identification methods.

To estimate the reactor temperature *y*(*k*) for input feed by the multilayer neural-based Hammerstein model structure,^7^ the network parameters are chosen as *p =* 3, *n*_a_ = 2, and *n*_b_=1, and hence, eq 6 can be written as
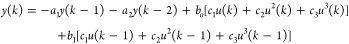
36

In the ELM-based Hammerstein model, the nonlinear static block of the Hammerstein model is an SLFN with five hidden neurons, and activation is a sigmoid function. The parameter vector as per eq 35 corresponds to the estimation of 12 parameters given as
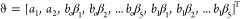
37

**Figure 7:**
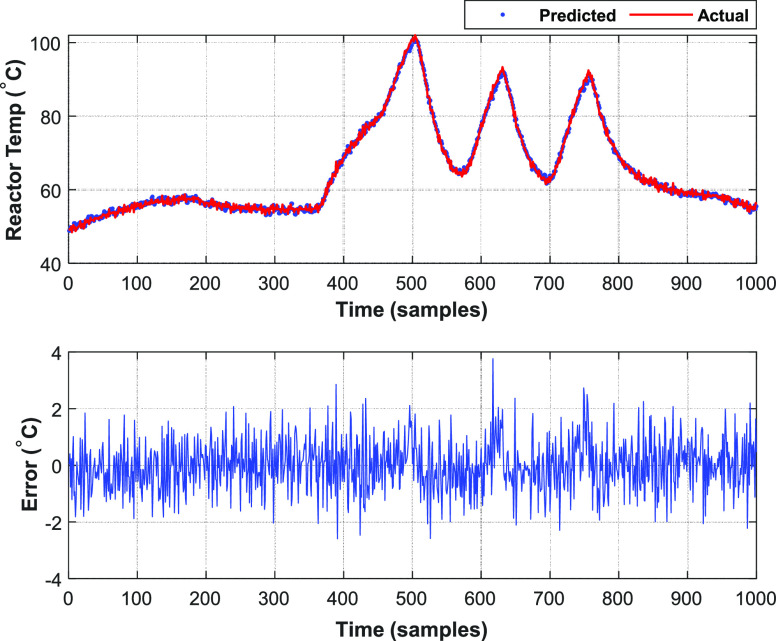
Multilayer NN-based Hammerstein model of the batch reactor.

**Figure 8:**
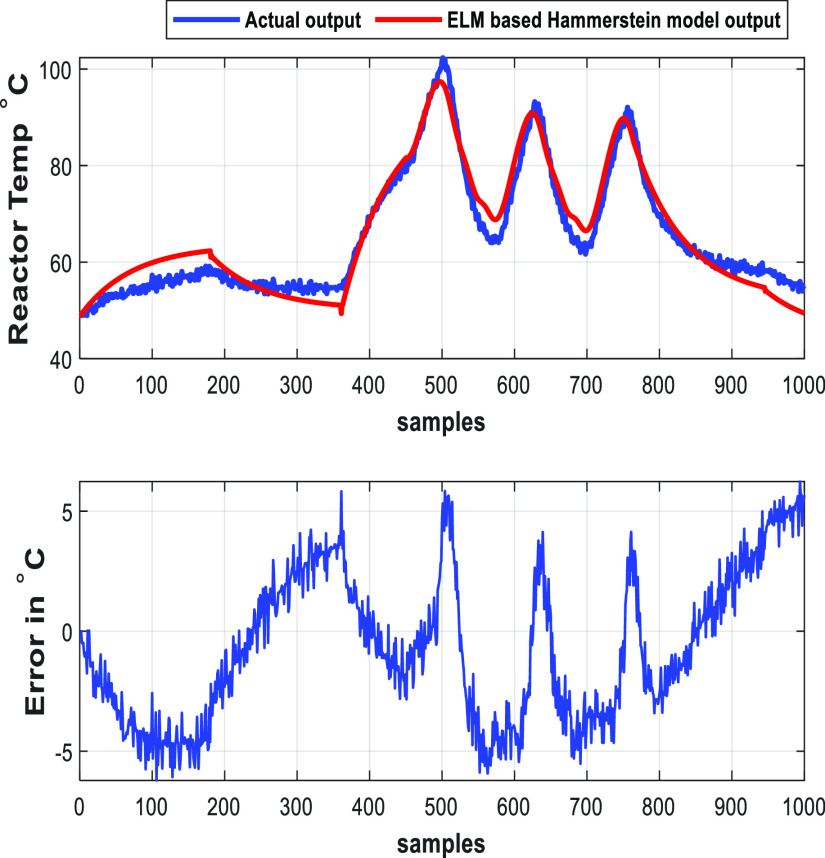
ELM-based Hammerstein model of the batch reactor.

**Figure 9:**
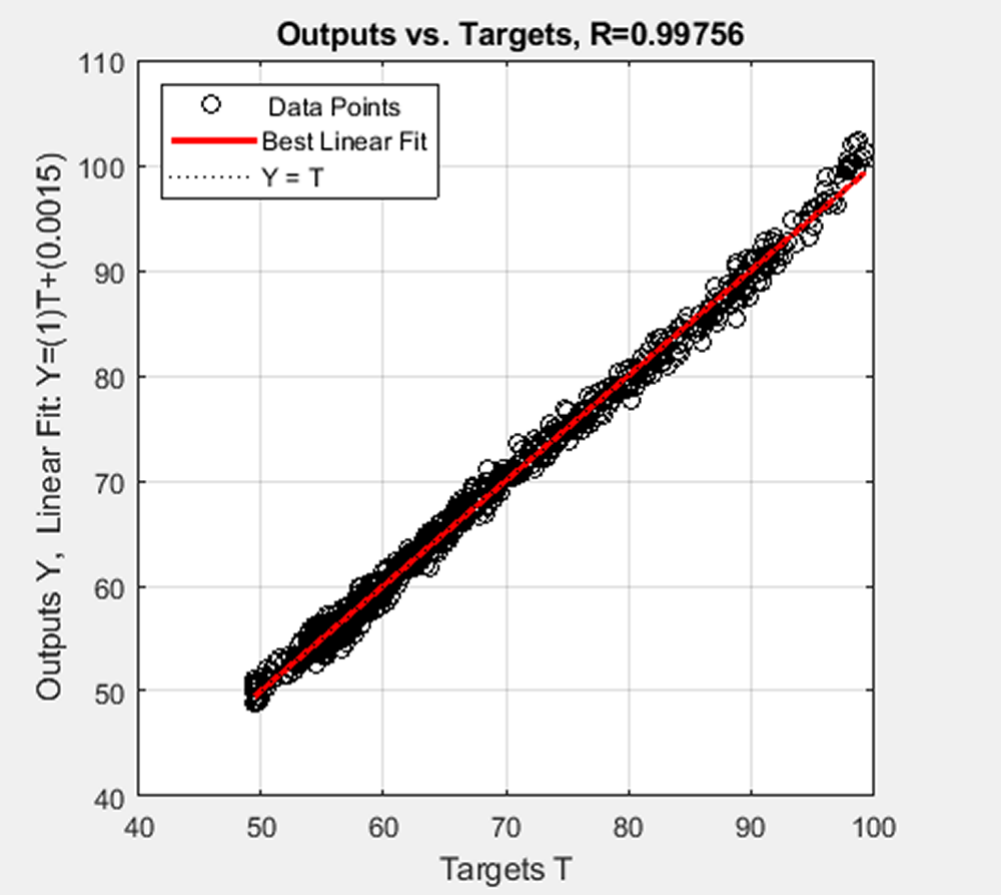
Regression fit of the multilayer neural-based Hammerstein model of the batch reactor.

**Figure 10:**
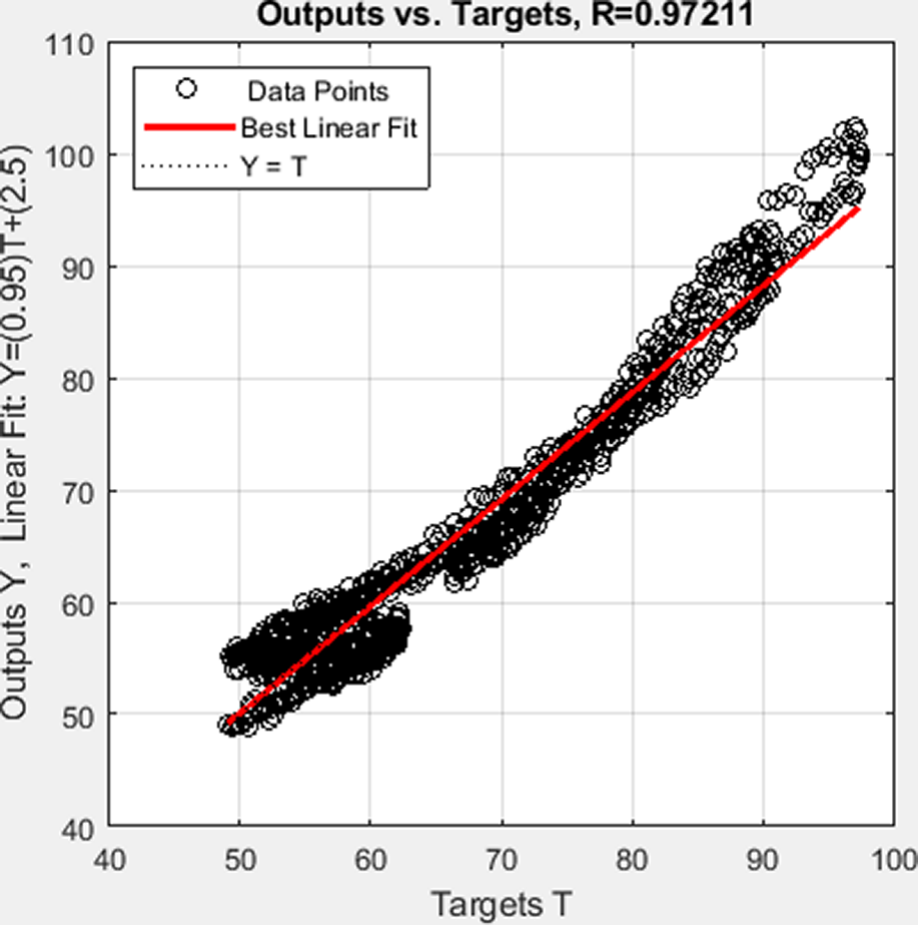
Regression fit of the ELM-based Hammerstein model of the batch reactor.

The reactor temperature identified by the multilayer neural-based Hammerstein model and ELM-based Hammerstein model are shown in Figures 7 and 8 and their regression fit is shown in Figures 9 and 10, respectively.

### 4.3 Performance Evaluation

To assess the performance of the identified neural-based Hammerstein models, the mean square error (MSE), mean absolute error (MAE), and root mean square error (RMSE) are found as a function of error *e_k_* between actual output from the plant data *y_k_* and predicted output employing corresponding model *ŷ*_*k*_ using eqs 38–40. The metrics are listed in Table 2:
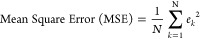
38
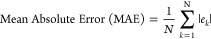
39
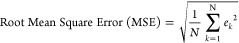
40

**Table 2:** Evaluation Metrics

evaluation metrics	multilayer neural-based Hammerstein model	ELM-based Hammerstein model
mean square error (MSE)	0.7839	9.5724
mean absolute error (MAE)	0.6835	2.6688
root mean square error (RMSE)	0.8853	3.0874
regression coefficient (*R*)	0.9976	0.9721

The inference from values of evaluation metrics in Table 2 identifies the multilayer neural-based Hammerstein model to be better than the ELM-based Hammerstein model. However, the computation time taken for the multilayer neural-based Hammerstein model is 92.58 s, whereas it is about 11.214 s for ELM-based Hammerstein model identification. The reason for less computation time of ELM-based identification is that it does not involve gradient calculation as backpropagation of weights is not involved in the algorithm.

## 5 Conclusions

Two types of Neural network-based Hammerstein models, namely, multilayer neural-based Hammerstein model and ELM-based Hammerstein model, are identified for a lab-scale batch reactor process. The first neural-based identification framed the entire Hammerstein model as a problem of gradient-based weight identification procedure for a multilayer network. The second type of identification is a gradient-free neural-based training and least-squares estimation of Hammerstein model parameters. Even though both the identification models are satisfactory in capturing the dynamics of the batch reactor process, the ELM-based Hammerstein model provides faster learning with less computations as backpropagation is not involved.

### 5.1 Future Studies

On the lab-scale batch reactor, a number of modeling approaches and nonlinear controllers were designed and implemented to track the ideal temperature profile. Currently, the modeling of the lab-scale batch reactor using various ML algorithms has been completed and the validation of ML-based nonlinear model predictive controller (NMPC) is in its final stage. Additionally, a batch reactor at the lab scale can also be used for the manufacture of a novel catalyst/biodiesel production. The optimal temperature profile for biodiesel production using deterministic and stochastic approaches was identified to use with ML-NMPC for closed-loop operation. Fuzzy-based ELM, reduced order Koopman modeling with MPC design for the batch reactor can also be carried out in the future.

**Figure 11:**
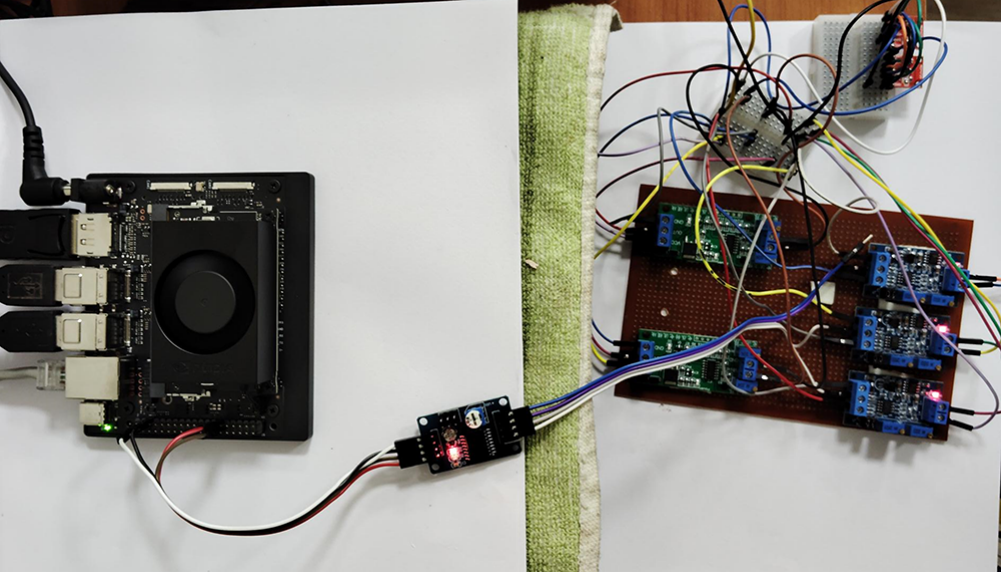
Jetson Orin 8GB Nano board interface with the lab-scale batch reactor for the machine learning-based nonlinear model predictive controller (NMPC) validation available in Research Lab-12, MIT, Manipal.

Figure 11 shows the Jetson Orin 8GB Nano board for the implementation of NMPC for the multilayer neural network-based Hammerstein model of the batch reactor. In recent past, the NMPC validated using MATLAB tool has limitations with the computing speed due to the online optimizer used in the NMPC. The usage of the Jetson Orin board will contribute toward the fast computation of control signals for the online computation and experimental validation for the highly nonlinear chemical process. In this case, the modeling of the batch reactor and controller part is coded using the Python 3.8 version. In order to connect the board with the pilot plant batch reactor, one needs an analog-to-digital converter and digital-to-analog converter along with the signal conditioning circuits.^20^ Sensors and actuators are connected via a GPIO pin configuration. This work is in its final stage to validate the ML-based NMPC algorithm on the pilot plant batch reactor.
